# Protective Role of Linagliptin in Cisplatin‐Mediated Liver Injury: Involvement of STAT3 and AMPK/SIRT1/PGC‐1alpha Mitochondrial Energy Sensing Networks

**DOI:** 10.1155/adpp/6457551

**Published:** 2026-07-07

**Authors:** Marawan A. Elbaset, Bassim M. S. A. Mohamed, Passant E. Moustafa, Yosra Assem Hussien, Zeinab A. El-Gendy, Sherif M. Afifi, Tuba Esatbeyoglu, Alyaa Farouk Hessin, Reda Korany M. S., Hany M. Fayed

**Affiliations:** ^1^ Department of Pharmacology, Medical Research and Clinical Studies Institute, National Research Centre, Giza, Egypt, nrc.sci.eg; ^2^ Department for Life Quality Studies, Rimini Campus, University of Bologna, Corso d’Augusto 237, Rimini, 47921, Italy, unibo.it; ^3^ Department of Molecular Food Chemistry and Food Development, Institute of Food and One Health, Gottfried Wilhelm Leibniz University Hannover, Am Kleinen Felde 30, Hannover, 30167, Germany, uni-hannover.de; ^4^ Department of Pathology, College of Veterinary Medicine, Cairo University, P.O. Box 12211, Cairo, Egypt, cu.edu.eg

**Keywords:** cisplatin, hepatoprotection, linagliptin, oxidative stress, SIRT1 signaling

## Abstract

Cisplatin (Cis), a commonly used chemotherapy drug, is associated with liver toxicity, which restricts its broader clinical use. This study investigated the potential protective effects of linagliptin (Lina), a DPP‐4 inhibitor, in preventing liver damage induced by Cis in rats. There were four groups of male rats: a control group, a Cis group (8 mg/kg, IP), and cotreated groups given Lina (5 and 10 mg/kg, orally) with Cis. Lina was administered daily for 15 days, with Cis injected on Day 8. Liver function, oxidative stress markers, inflammatory mediators, energy metabolism indicators, and key signaling proteins were assessed. Cis administration resulted in significant hepatotoxicity, evidenced by elevated liver enzymes, increased oxidative stress, enhanced inflammatory response, and disrupted energy metabolism. Lina treatment, particularly at the 10‐mg/kg dose, demonstrated marked hepatoprotective effects. It significantly reduced liver enzyme levels, improved antioxidant status, attenuated inflammatory markers, and restored energy metabolism indicators. Moreover, Lina positively modulated essential signaling proteins involved in cellular stress response and metabolism, including signal transducer and activator of transcription 3 (STAT3), transforming growth factor beta 1 (TGF‐β), silent information regulator 1 (SIRT1), and peroxisome proliferator–activated receptor gamma coactivator 1‐alpha (PGC‐1α). The results indicate that Lina protects against Cis‐induced liver damage by leveraging its antioxidant, anti‐inflammatory, and metabolic regulation properties. This study offers new insights into potential strategies for mitigating Cis‐induced hepatotoxicity and enhancing its therapeutic index in cancer treatment.

## 1. Introduction

Cisplatin (Cis) is a highly effective and widely used chemotherapeutic agent for the treatment of various malignancies, including cancers of the testes, ovaries, cervix, bladder, as well as those affecting the head, neck, and lungs [1]. Despite its clinical efficacy, its use is significantly limited by a range of dose‐dependent toxicities, most notably nephrotoxicity and hepatotoxicity, along with cardiotoxicity, neurotoxicity, and ototoxicity [2]. As a xenobiotic, Cis is metabolized and detoxified primarily in the liver [3]. Its accumulation in hepatic tissue disrupts cellular redox balance, leading to elevated oxidative stress and the subsequent initiation of inflammatory cascades [4]. The overproduction of reactive oxygen species (ROS), including superoxide anions and hydroxyl radicals, promotes lipid peroxidation and consequent cellular injury [3]. Furthermore, increased intracellular ROS levels are closely linked to the activation of the nuclear factor kappa B (NF‐κB) signaling pathway, a key regulator of proinflammatory cytokine synthesis [5–7]. This activation subsequently upregulates the expression of inflammatory mediators such as tumor necrosis factor‐alpha (TNF‐α) and interleukin‐6 (IL‐6), exacerbating liver damage [8]. Given that oxidative stress is a primary mechanism underlying Cis‐induced hepatotoxicity, antioxidant‐based interventions have shown promise in mitigating this adverse effect [9, 10].

Silent information regulator 1 (SIRT1) is a cytoprotective protein that, when activated, counteracts the toxicity of Cis [11]. SIRT1 is involved in many biological processes [12]. Further, SIRT1 can shield cells from harm and promote cell survival under biological stress [13]. By deacetylating several signaling molecules, such as NF‐κB and SIRT1, tissue damage and inflammation can be prevented [14, 15]. PGC‐1α, or peroxisome proliferator–activated receptor‐gamma coactivator, is a group of transcriptional cofactors that regulates the production of proteins essential for mitochondrial function [16]. It has been demonstrated that PGC‐1α can cooperatively activate several nuclear receptors and transcription factors linked to oxidative stress, such as nuclear respiratory factor 1 (NRF1) [17]. SIRT1 works in conjunction with histone acetylase (GCN5) to control the transcriptional activity of PGC‐1α [18].

Linagliptin (Lina) is a potent, selective, and long‐acting inhibitor of dipeptidyl peptidase‐4 (DPP‐4), clinically approved for the management of Type 2 diabetes mellitus. It functions by prolonging the activity of incretin hormones, thereby enhancing insulin secretion and lowering blood glucose levels [19]. Lina, a DPP‐4 inhibitor approved for Type 2 diabetes, has demonstrated antitumor effects in preclinical models. It inhibits cancer cell growth and triggers cell death by modulating critical signaling pathways, such as PI3K/AKT and ERK, that are essential for tumor cell survival [20]. Recent studies have demonstrated that Lina exerts protective effects beyond glycemic control. In normoglycemic animal models, Lina has been shown to ameliorate testicular damage [21], chronic kidney injury [22], and inflammatory bowel disease [23]. These protective effects are attributed to its antioxidant, anti‐inflammatory, and antiapoptotic properties, partially mediated through AMPK activation [22] and modulation of the SIRT1/PGC‐1*α* pathway [23]. Regarding hepatoprotection specifically, Aboulmagd et al. [24] demonstrated that Lina prevented the progression of hepatic fibrosis in diabetic obese rats, while Almalki et al. [25] recently reported its protective effects against thioacetamide‐induced liver fibrosis. However, to date, no study has investigated whether Lina can protect against the acute hepatotoxicity induced by Cis chemotherapy. Therefore, the current study aimed to evaluate, for the first time, Lina’s potential to protect rats from Cis‐induced hepatotoxicity and to elucidate the underlying mechanisms, with a particular focus on the SIRT1/PGC‐1α mitochondrial energy‐sensing network and the STAT3 signaling pathway. This investigation is novel in three aspects: (1) it is the first to examine Lina in the context of Cis‐induced hepatotoxicity; (2) it comprehensively evaluates the AMPK/SIRT1/PGC‐1α axis in this specific model; and (3) it explores the interplay between STAT3 and mitochondrial energy sensors in mediating Lina’s hepatoprotective effects. By addressing these gaps, this study provides novel insights into the therapeutic potential of Lina as an adjuvant therapy to mitigate the hepatic side effects of Cis chemotherapy, paving the way for future translational research.

## 2. Material and Methods

### 2.1. Drugs and Chemicals

A parenteral vial of Cis was purchased from EIMC Pharmaceuticals Company, Cairo, Egypt, and Lina (Trajenta) was purchased from Boehringer Ingelheim Roxane Inc., Columbus, Ohio, USA.

### 2.2. Lab Animals

The National Research Centre (NRC) at Egypt’s Animal House Colony provided twenty‐four Wistar albino rats, each weighing between 180 and 200 g. A 12‐h light/dark cycle was applied to them, and they were maintained at a consistent temperature of 25 ± 2°C, provided with a standard pellet diet, and had unlimited access to water. All procedures involving animal handling followed the guidelines set by the Research Ethical Committee of the NRC, Egypt, with ethical approval number 01450124.

### 2.3. Study Design

Rats underwent a one‐week acclimation period before the trial. A random division into four groups was then applied (*n* = 6). Group 1 served as the control group. Group 2, which served as the Cis toxic control, received a single dose of Cis (8 mg/kg, IP) on the eighth day of the research [26]. Group 3 (Lina + Cis) was treated with Lina at an oral dose of 5 mg/kg, whereas Group 4 (Lina + Cis) received Lina at an oral dose of 10 mg/kg [27]. Lina was administered daily to Groups 3 and 4 (the treatment groups) throughout the 15‐day experimental period, and the Lina groups received Cis on Day 8 of the experiment, following the same schedule as Group 2 (Cis control).

#### 2.3.1. Blood and Tissue Collection

Rats were sedated with ketamine 50 mg/kg and xylazine 25 mg/kg at the end of the research period, and blood samples were taken from the tail vein before they were sacrificed by cervical dislocation. Serum samples were stored at −20°C until they were needed for biochemical analysis. Following direct extraction, the liver tissues were rinsed in ice‐cold saline and then divided into sections. For molecular studies, a piece of the liver from each weighed rat was stored at −80°C. The histology and immunohistochemistry sections were kept apart in 10% buffered neutral formalin.

### 2.4. Evaluations of Biochemistry

#### 2.4.1. Hepatic Function and Lipid Metabolism Biomarkers

Serum concentrations of alanine aminotransferase (ALT) and aspartate aminotransferase (AST) (Biomatik, USA; Cat# EKE62019 and Cat# EKU02211, respectively) were measured to evaluate liver injury [28]. Total cholesterol (TC) and triacylglycerol (TAG) levels in serum were analyzed following the protocols provided by Spectrum (Cairo, Egypt) and Cayman (USA; Cat#1001302).

#### 2.4.2. Oxidative Stress Indicators

Liver homogenates were tested for malondialdehyde (MDA), a sign of lipid peroxidation, using the “BioVision” methodology (Milpitas Boulevard, Milpitas, USA; “Cat# K739‐100”) [29]. Additionally, in accordance with the guidelines provided by the manufacturers, the superoxide dismutase (SOD) BT‐LAB kit (Cat# E1444Ra, Shanghai, China) [30] and the glutathione (GSH) BioVision kit (Cat# K464‐100, Milpitas Boulevard, USA) [31] were used to assess SOD and GSH in hepatic tissue using colorimetric kits.

#### 2.4.3. Inflammatory Biomarkers

TNF‐α and IL‐6 levels were measured according to the manufacturer’s protocols from BioLegend (San Diego, USA; Cat# 438204) and CLOUD‐CLONE CORP (USA; Cat# SEA079Ra), respectively. Additionally, the protocols for interleukin‐10 (IL‐10), p‐NF‐κB, and p‐STAT‐3 were provided by CLOUD‐CLONE CORP. (USA; Cat# SEA065Ra), Sunlong (China; Cat# SLD1755Ra), and AFG Scientific (USA; Cat# EK721641).

#### 2.4.4. Measurement of Adenosine Triphosphate (ATP)/Adenosine Diphosphate (ADP) and Nicotinamide adenine (NADP/NADPH)

Levels of ATP/ADP and NADP/NADPH were assessed using the ELISA method in liver tissues, following the manufacturer’s protocol for ATP/ADP (Abcam, Cambridge, UK; Cat# ab65313) and NADP/NADPH (Abcam, Cambridge, UK; Cat# ab65349).

#### 2.4.5. Western Blotting

An EDTA‐free protease inhibitor cocktail in 10 mM Tris‐HCl, 100 mM NaCl, 0.5% Triton X‐100, and pH 7.6 was used to sonicate and lyse the tissue. The Pierce BCA Protein Assay Kit (Thermo Fisher Scientific) was used to quantify the protein, and 25 μg of protein was subjected to sodium dodecyl sulfate‐polyacrylamide gel (SDS‐PAGE). The primary antibodies used in the Western blot analysis were procured from Elabscience, Texas, USA and SANTA CRUZ BIOTECHNOLOGY, Inc., Oregon, USA. They were identified by catalog numbers E‐AB‐32901 and (4A8): sc‐517380, respectively, to identify SIRT1 and PGC1‐α [32].

#### 2.4.6. Examination of Liver Tissue Histology

Samples of liver tissue were obtained from each group after the experiment, preserved in 10% neutral buffered formalin, cleaned, dried, and embedded in paraffin. After being sectioned to a 5‐micron thickness, the paraffin‐embedded blocks were stained with H&E for histological analysis [33]. Using a light microscope, the stained tissue sections were viewed (Olympus BX50, Japan).

#### 2.4.7. Scoring of Histopathological Lesions

Following the recording of liver histological alterations, scores of 0 (*no changes*), 1 (*mild*), 2 (*moderate*), or 3 (*severe*) were assigned. Grades were assigned according to the percentage of changes: mild (less than 30%), moderate (30%–50%), and severe (more than 50%).

### 2.5. Immunohistochemistry

To prepare liver tissue sections, the samples were deparaffinized with xylene and rehydrated using a series of alcohol concentrations. For 20 min, the sections were incubated in citrate buffer at pH 6 to retrieve the antigen. The sections were then treated with mouse monoclonal alpha‐smooth muscle actin (α‐SMA) antibody (M0851; 1:100 dilutions, DAKO, Santa Clara, US) and rabbit monoclonal TGF‐β1 (ab215715; 1:500 dilutions, Abcam, Cambridge, UK) for two hours in a humidified atmosphere. Next, goat antirabbit IgG H&L (HRP) (ab205718; Abcam, Cambridge, UK) was combined with 3,3′‐diaminobenzidine tetrahydrochloride (DAB; Sigma) as the chromogen. Hematoxylin was used as a counterstain, and DPX was used to mount the slides. PBS was substituted for the primary antibodies to establish negative controls.

#### 2.5.1. Evaluation of α‐SMA and TGF‐β1 Immunostaining

The quantitative immunoreactivity of TGF‐β1 and α‐SMA was assessed in tissue sections from each group, as described by Alshafei et al. [34]. Five tissue sections were analyzed, with immunoreactivity evaluated in 10 microscopic fields per section at high magnification (× 400). Color deconvolution was used with ImageJ 1.52p software (Wayne Rasband, National Institutes of Health, USA) to calculate the proportion of positively stained cells.

### 2.6. Statistical Analysis

The mean ± SEM is used to display the data. To do statistical analyses, GraphPad Prism (Version 6.0; San Diego, CA, USA) was used. The Tukey–Kramer post hoc test was used after analysis of variance (ANOVA) to assess multiple comparisons. *p* values below 0.05 were regarded as statistically significant.

## 3. Results

### 3.1. Effect of Lina in Modulating Liver Enzyme Levels: AST and ALT in Rats With Cis Toxicity

Cis (8 mg/kg) administration resulted in significant liver damage, as evidenced by elevated liver enzymes. AST and ALT levels increased by 214% and 127%, respectively, compared to the normal group. Lina treatment at 5 and 10 mg/kg doses showed hepatoprotective effects, reducing AST levels by 39% and 69% and ALT levels by 26% and 49%, compared with the Cis group. The 10‐mg/kg dose appeared to restore these enzymes to near‐normal levels (Figure 1).

**FIGURE 1:**
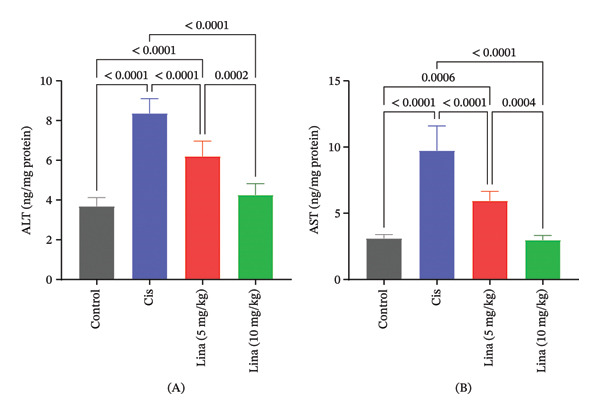
Assessment of linagliptin’s impact on liver function: AST and ALT in cisplatin‐treated rats. (A) Serum ALT (U/L) and (B) serum AST (U/L). Data are presented as mean ± SEM of six rats, with *p* values displayed on the bars. Cis: cisplatin; lina: linagliptin.

### 3.2. Effect of Lina on Triglycerides and TC Levels in Cis‐Induced Toxicity in Rats

Lipid profile markers were also negatively impacted by Cis, with triglycerides and TC increasing by 156% and 98%, respectively. Lina treatment at 5 and 10 mg/kg reduced triglyceride levels by 21% and 54% and TC by 2.5% and 41%, compared to the Cis rats. The 10‐mg/kg dose displayed more pronounced improvement, bringing these markers closer to normal levels (Figure 2).

**FIGURE 2:**
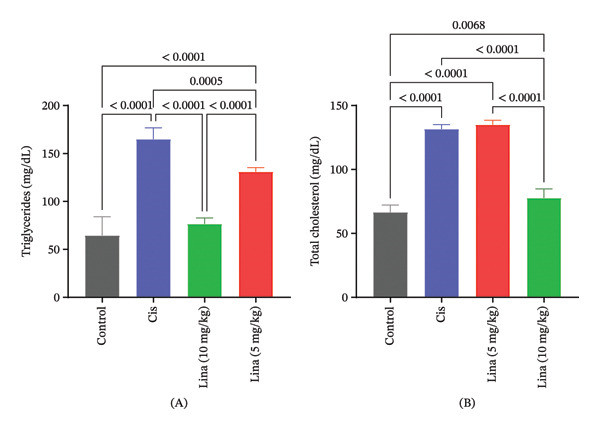
Assessment of linagliptin’s effect on lipid profile: serum triglycerides and total cholesterol in cisplatin‐treated rats. (A) Serum triglycerides (mg/dL) and (B) serum total cholesterol (mg/dL). Results are shown as mean ± SEM for six rats, with exact *p* values indicated by the bars. Cis: cisplatin; lina: linagliptin.

### 3.3. Effect of Lina on Oxidation Stress: GSH, SOD, and MDA in Cis‐Intoxicated Rats

Oxidative stress markers were significantly affected by Cis treatment compared to the control group. GSH and SOD levels decreased by 65% and 75%, respectively. In contrast, MDA increased by 378% compared to the normal group. Lina treatment showed antioxidant effects, with the 10‐mg/kg dose increasing GSH by 174% and SOD by 254% and declining MDA by 76% compared to the Cis group, nearly restoring these markers to normal levels (Figure 3).

**FIGURE 3:**
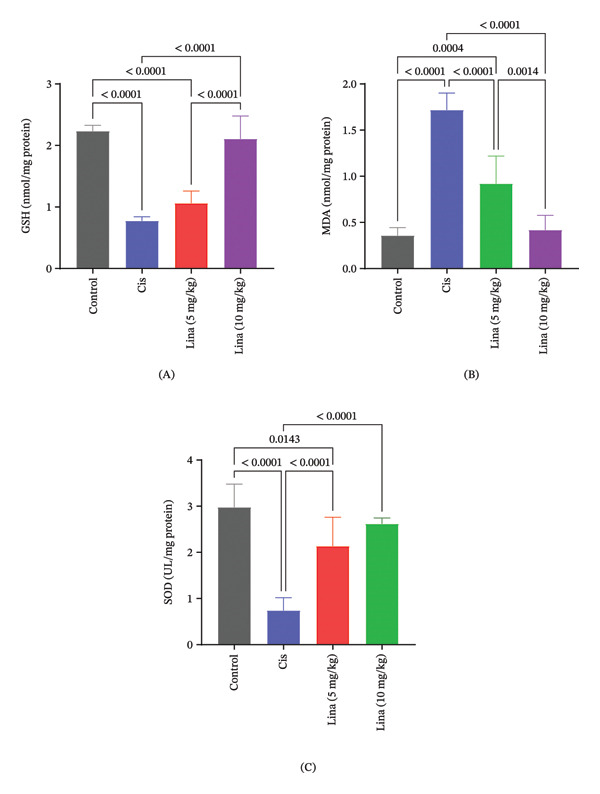
Linagliptin’s effect on oxidative stress markers: GSH, SOD, and MDA in rats with cisplatin‐induced toxicity. (A) GSH (nmol/mg protein), (B) SOD (U/mg protein) activity, and (C) MDA (nmol/mg protein). Each bar represents the mean ± SEM of six rats; the exact *p* values are indicated by the bars. Cis: cisplatin; lina: linagliptin.

### 3.4. Effect of Lina on Inflammatory Mediators: TNF‐α, IL‐6, IL‐10, and NF‐κB in Cis‐Intoxicated Rats

Inflammatory markers were markedly elevated in the Cis group. TNF‐α, IL‐6, and p‐NF‐κB increased by 292%, 216%, and 260%, respectively, while the anti‐inflammatory IL‐10 decreased by 26% compared to the control group. Lina treatment, particularly at 10 mg/kg, showed anti‐inflammatory effects, reducing TNF‐α by 63%, IL‐6 by 61%, and p‐NF‐κB by 63%, while increasing IL‐10 by 23% compared to the Cis group, bringing these markers closer to normal levels (Figure 4).

**FIGURE 4:**
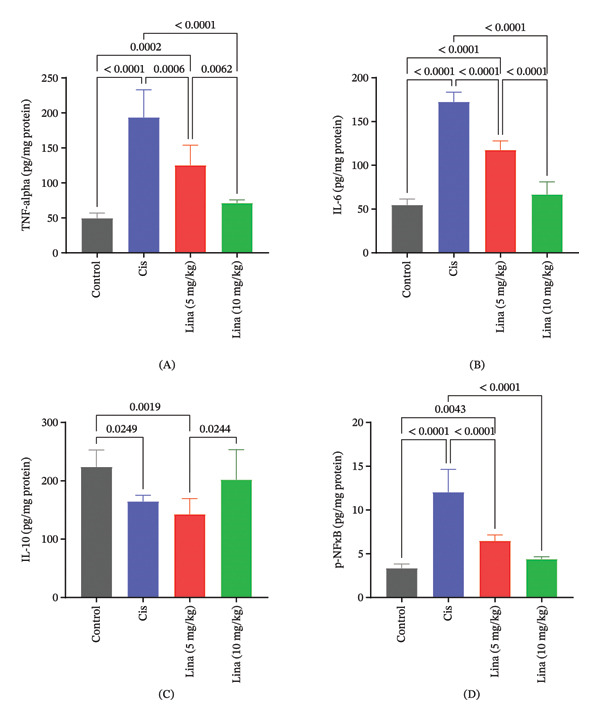
Effect of linagliptin on inflammatory mediators: TNF‐α, IL‐6, IL‐10, and NF‐κB in cisplatin‐intoxicated rats. (A) TNF‐α (pg/mg protein), (B) IL‐6 (pg/mg protein), and (C) IL‐10 (pg/mg protein), (D) NF‐κB (pg/mg protein). Each bar represents the mean ± SEM of six rats; the exact *p* values are indicated by the bars. Cis: cisplatin; lina: linagliptin.

### 3.5. Effect of Lina on Energy Metabolism Indicators: ADP and ATP in Cis‐Intoxicated Rats

Energy metabolism indicators ADP and ATP were disrupted by Cis, increasing by 282% and 214%, respectively, compared to the control group. Lina treatment at 10 mg/kg reduced ADP by 58% and ATP by 64% compared to the Cis group, suggesting improved energy metabolism. The ADP/ATP ratio, while not directly calculable from the given data, appeared to be partially normalized by Lina treatment (Figure 5).

**FIGURE 5:**
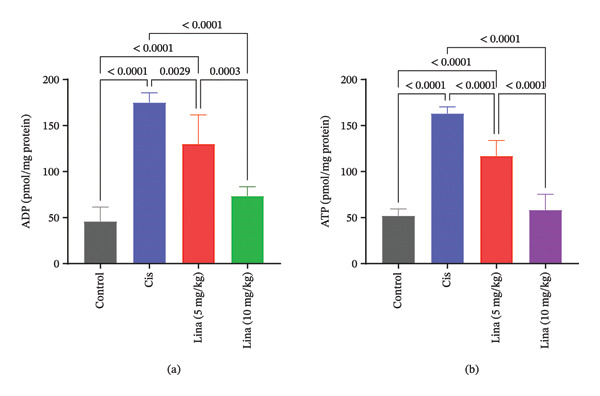
Effect of linagliptin on energy metabolism indicators: ADP and ATP in cisplatin‐intoxicated rats. (a) ADP (pmol/mg protein) and (b) ATP (pmol/mg protein). Each bar represents the mean ± SEM of six rats; the exact *p* values are indicated by the bars. Cis: cisplatin; lina: linagliptin.

### 3.6. Effect of Lina on Redox Status: NADP and NADPH in Cis‐Intoxicated Rats

NADP levels decreased by 61% and NADPH by 69% in the Cis group compared to the control. Lina treatment at 10 mg/kg increased NADP by 152% and NADPH by 171% compared to the Cis group, nearly restoring these levels to normal. The NADP/NADPH ratio, though not directly calculable, appeared to be improved by Lina treatment (Figure 6).

**FIGURE 6:**
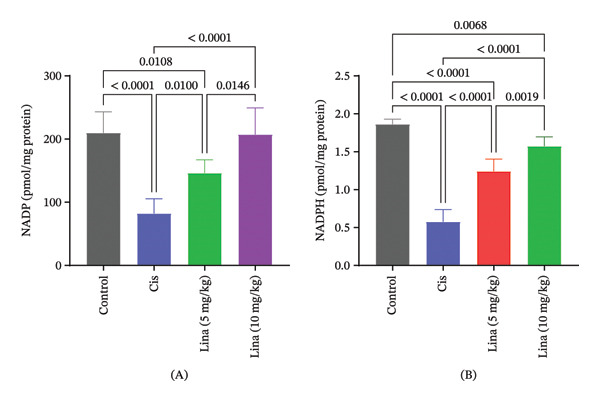
Effect of linagliptin on redox status: NADP and NADPH in cisplatin‐intoxicated rats. (A) NADP (pmol/mg protein) and (B) NADPH (pmol/mg protein). Each bar represents the mean ± SEM of six rats; the exact *p* values are indicated by the bars. Cis: cisplatin; lina: linagliptin.

### 3.7. Effect of Lina on p‐STAT3 in Cis‐Intoxicated Rats

The p‐STAT3 level was significantly increased in the Cis group by 215%, compared to the control group. Lina treatment, especially at 10 mg/kg, showed improvements in this marker, reducing p‐STAT3 by 57% compared to the Cis group (Figure 7).

**FIGURE 7:**
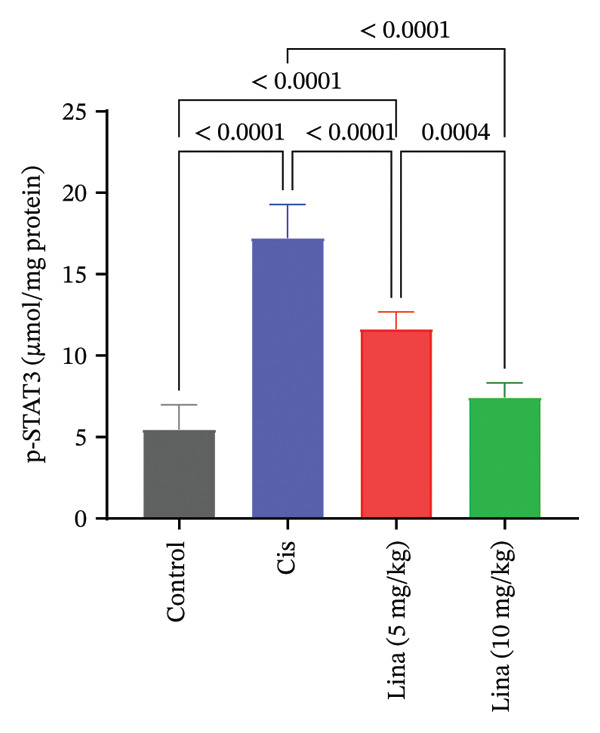
Effect of linagliptin on p‐STAT3 level in cisplatin intoxicated rats. Each bar represents the mean ± SEM of six rats; the exact *p* values are indicated by the bars. Cis: cisplatin; lina: linagliptin.

### 3.8. Effect of Lina on Signaling Proteins: Sirt‐1 and PGC‐1α in Cis‐Intoxicated Rats

Signaling proteins Sirt‐1 and PGC‐1α were negatively affected by Cis. Compared with the control, Sirt‐1 and PGC‐1α decreased by 83% and 86%, respectively. Lina treatment, especially at 10 mg/kg, showed improvements in these markers, increasing Sirt‐1 by 344% and PGC‐1α by 500% compared to the Cis group, indicating the potential restoration of normal signaling pathways (Figure 8).

**FIGURE 8:**
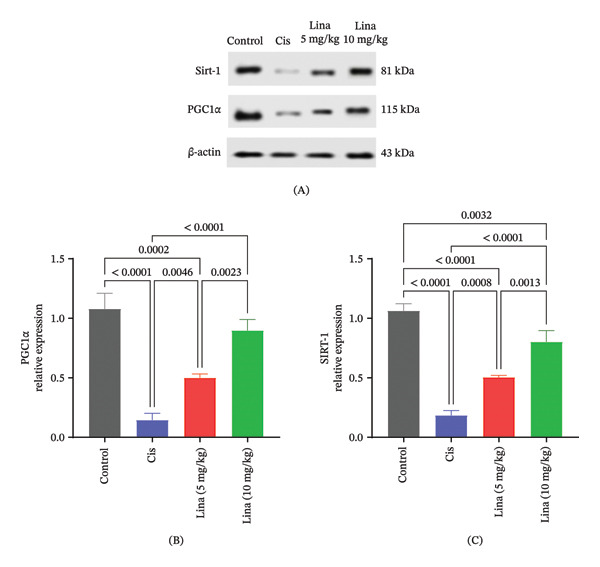
Western blot of the effect of linagliptin on signaling proteins SIRT‐1 and PGC‐1α in cisplatin‐intoxicated rats. (A) Western blot (B) PGC‐1α and (C) SIRT‐1. Each bar represents the mean ± SEM of three experiments; the exact *p* values are indicated by the bars. Cis: cisplatin; lina: linagliptin.

### 3.9. Histopathological Findings

The control group’s hepatocyte histological structure was normal (Figure 9a). Most hepatocytes in the Cis group displayed severe vacuolar degeneration (Figure 9b), while the portal area displayed blood vessel congestion, bile duct hyperplasia, and portal fibrosis (Figure 9c). A small percentage of these hepatocytes also displayed coagulative necrosis (Figure 9d). Significant hepatocyte vacuolar degeneration (Figure 9e) and mild portal fibrosis (Figure 9f) were observed in the Lina group (5 mg/kg). The Lina group (10 mg/kg) revealed nearly normal hepatocytes and the portal area (Figure 9g,h).

**FIGURE 9:**
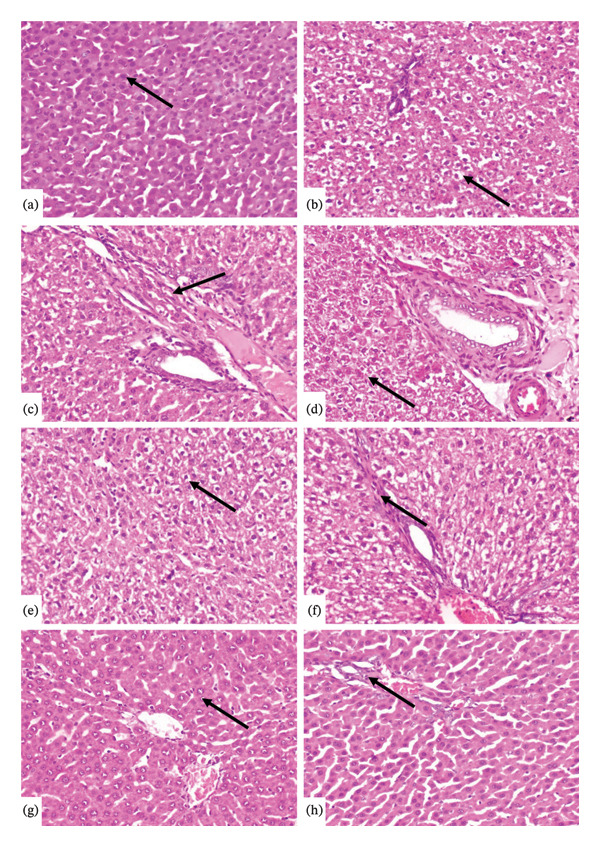
Photomicrograph, rat liver (H&E‐stained sections), (a) control group showing the normal histological structure of hepatocytes (arrow). (b) The group treated with cisplatin showed severe vacuolar degeneration of most hepatocytes (arrow). (c) The group treated with cisplatin showed hyperplasia of the bile duct and portal fibrosis (arrow). (d) The group treated with cisplatin showed coagulative necrosis of some hepatocytes (arrow). (e) The group treated with linagliptin (5 mg/kg) showed moderate vacuolar hepatocyte degeneration. (f) The group treated with linagliptin (5 mg/kg) showing mild portal fibrosis (arrow). (g) The group treated with linagliptin (10 mg/kg) showing normal hepatocytes (arrow). (h) The group treated with linagliptin (10 mg/kg) showed the normal portal area (arrow) (× 400).

#### 3.9.1. Scoring of the Hepatic Histopathological Lesion

Table 1 illustrates the severity scores assigned to all liver lesions that were documented.

**TABLE 1:** Histopathological alterations scoring in the liver of treated groups.

Lesions	Control	Cis	Lina (5 mg/kg)	Lina (10 mg/kg)
Vacuolar degeneration of hepatocytes	0	3	2	0
Coagulative necrosis of hepatocytes	0	3	1	0
Portal fibrosis	0	3	1	1
Hyperplasia of bile ducts	0	2	1	1

*Note:* The scoring system was established as Score 0 = no lesions observed in any rats of the group (*n* = 5); Score 1 = lesions in less than 30% of rats; Score 2 = lesions in 30%–50% of rats; Score 3 = lesions in more than 50% of rats.

### 3.10. Immunohistochemical Findings of TGF‐β1and α‐SMA in the Liver

Immunostaining expression of TGF‐β1and α‐SMA % area in liver tissue of different experimental groups is illustrated in Figure 10. The control group showed very weak immune expression of TGF‐β1 in hepatocytes and α‐SMA in the portal area (Figure 10a,b). The Cis group displayed strong immunoreactivity for TGF‐β1 in hepatocytes and α‐SMA in the portal area (Figure 10c,d). The Lina group (5 mg/kg) demonstrated a decrease in the immune‐staining response of α‐SMA in the portal region and TGF‐β1 in hepatocytes (Figure 10e,f). The Lina group (10 mg/kg) showed weak immune‐reactive cells of TGF‐β1 in hepatocytes and α‐SMA in the portal area (Figure 10g,h).

FIGURE 10Photomicrograph, rat liver (TGF‐β1 and α‐SMA stained sections), (a and b) control group showing very weak immune expression of TGF‐β1 in hepatocytes and α‐SMA in the portal area. (c and d) Group treated with cisplatin showing strong immunoreactivity of TGF‐β1 in hepatocytes and α‐SMA in the portal area. (e and f) Group treated with linagliptin (5 mg/kg) showing significant reduction in the immune‐staining reaction of TGF‐β1 in hepatocytes and α‐SMA in the portal area. (g and h) Group treated with linagliptin (10 mg/kg) showing weak immune‐reactive cells of TGF‐β1 in hepatocytes and α‐SMA in the portal area (× 400). (i) Percentage area of TGF‐β1 expression in various treatment groups (results expressed as mean ± SE, with distinct letters indicating significant differences at *p* < 0.05). (j) Percentage area of α‐SMA expression in various treatment groups (data presented as mean ± SE, with different letters indicating significant differences at “*p* < 0.05)”.

**Figure:**
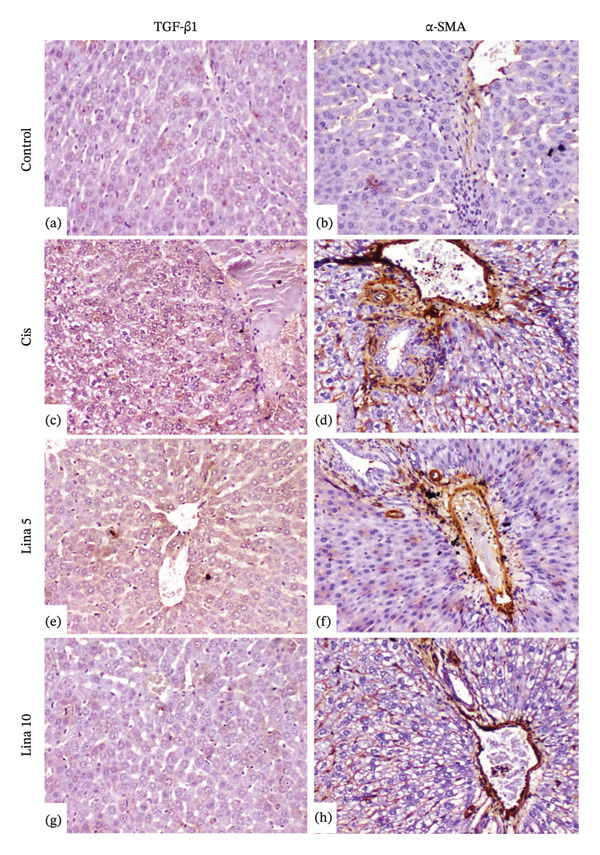

**Figure:**
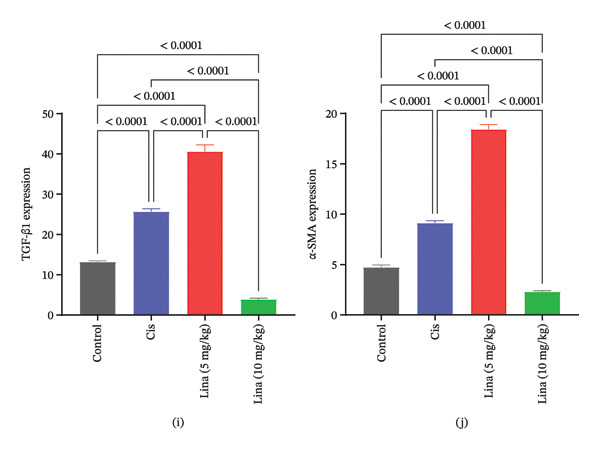

## 4. Discussion

Cis, a chemotherapeutic agent employed in the treatment of solid organ tumors, is associated with adverse effects across multiple organs, including hepatotoxicity. Our findings indicated that Cis can markedly induce hepatic toxicity in rats, as indicated by elevated serum liver enzyme activities, increased oxidative stress, inflammatory responses, mitochondrial dysfunction, and histopathological changes [35, 36]. This study utilized a rat model of Cis‐induced hepatotoxicity to investigate the possible mechanisms by which Lina mitigates liver damage. The study findings revealed that Lina had a multifaceted impact, mitigating liver toxicity by improving liver enzyme levels, as evidenced by decreased levels of ALT and AST. Additionally, Lina alleviated oxidative stress by boosting antioxidant defenses, demonstrated by increased SOD and GSH levels and reduced lipid peroxidation, as indicated by decreased MDA content. Moreover, Lina suppressed inflammatory responses, as shown by elevated IL‐10 levels and reduced TNF‐α, p‐NF‐κB, IL‐6, and TGFβ1 contents. It also improved mitochondrial function, reflected in the restoration of ADP/ATP and NADP/NADPH ratios, both of which are crucial for cellular energy metabolism. The observed protective effects are associated with the modulation of the STAT‐3/SIRT1/AMPK/PGC1α signaling pathway. Lina treatment notably enhanced the immunoreactivity of SIRT1, PGC1α, and p‐STAT‐3 in liver tissue. To our knowledge, this is the first research to investigate the impact of Lina on Cis‐induced liver injury in rats via this specific pathway. The modulation of the IL‐6/STAT‐3 and SIRT1/AMPK/PGC1α axes appears to be crucial for the observed antioxidant and anti‐inflammatory effects, as well as for the improvement in mitochondrial function.

A single dose of Cis (8 mg/kg) in this investigation resulted in hepatotoxicity, as shown by substantial elevations in liver enzyme markers, particularly serum ALT and AST, and disturbances in lipid metabolism, including high levels of TAG and TC. These outcomes align with those of other investigations [37–39], which suggest that these changes are anticipated after rats receive Cis. In contrast, Lina (5 and 10 mg/kg) therapy decreased serum ALT and AST levels compared to untreated rats, thereby mitigating the hepatotoxic consequences of experimentally induced liver damage. Comparing Lina‐treated rats to those that were not, the former showed a significant decrease in serum TAG and TC levels. These findings collectively underscore the hepatoprotective effects of Lina and align with the findings in [24, 25], indicating its impact in alleviating liver damage.

At this point, we initiated an investigation into the underlying mechanisms driving the antioxidant and anti‐inflammatory effects of Lina. In the present study, a single dose of Cis (8 mg/kg) induced hepatotoxicity, leading to decreases in GSH, SOD, and IL‐10 tissue contents, while causing increases in MDA, TNF‐α, IL‐6, TGFβ, and p‐NF‐κB. Supporting our study, evidence indicates that Cis administration notably increases oxidative stress and inflammation in liver tissue, causing both histopathological damage and functional alterations [40]. Treatment with Lina (5 and 10 mg/kg) demonstrated significant antioxidant and anti‐inflammatory effects, evidenced by increases in GSH, SOD, and IL‐10 tissue contents, alongside reductions in MDA, TNF‐α, IL‐6, TGFβ, and p‐NF‐κB. These results are consistent with earlier research suggesting that DPP‐4 inhibitors, or gliptins, have hepatoprotective effects owing to their capacity to reduce oxidative stress and inflammation [24, 41, 42].

The current investigation has shown that Cis (8 mg/kg) impairs mitochondrial functions, as indicated by decreases in ATP, ADP, NADP, and NADPH contents. Consistent with our findings, previous studies have reported that Cis impairs mitochondrial functions through several mechanisms. It causes oxidative stress, which results in the production of ROS that harm DNA, proteins, and mitochondrial membranes. This damage inhibits critical enzymes in the electron transport chain, leading to reduced ATP production. Furthermore, Cis disrupts the citric acid cycle and depletes essential cofactors such as NADP and NADPH, further impairing mitochondrial energy metabolism and exacerbating oxidative stress. These effects result in decreased levels of ATP, ADP, NADP, and NADPH, indicating substantial mitochondrial dysfunction [43, 44]. According to our findings, Lina (5 and 10 mg/kg) significantly increased the expression of SIRT1, which is crucial for maintaining ATP balance during energy metabolism. Through the activation of pathways that increase ATP synthesis and decrease energy expenditure, in coordination with SIRT1, p‐AMPK regulates cellular energy homeostasis [45, 46]. Specifically, SIRT1 maintains ATP levels by controlling metabolic activities and mitochondrial function [47, 48]. The fact that Lina has upregulated these proteins suggests that energy metabolism has been positively impacted, stabilizing ATP levels and enhancing general cellular performance. Our results are consistent with those of El‐Ghannam et al. [23], who reported that Lina treatment significantly upregulated SIRT1 gene expression.

PGC‐1α, a coactivator and transcription factor, is essential for regulating mitochondrial biogenesis and maintenance. Conversely, p‐STAT‐3, which increases in response to liver damage, has double‐edged roles as it may play a crucial role in reducing inflammation and promoting cell survival and repair; on the other hand, several investigations demonstrated that STAT3 is involved in fibrosis by stimulating the production of the extracellular matrix [23, 49–51]. It has been observed that Cis (8 mg/kg) decreased PGC‐1*α* and increased p‐STAT‐3 contents in the liver tissue of rats. These findings are consistent with the results reported in several studies [49, 52]. In the current investigation, Lina therapy resulted in elevated levels of PGC‐1*α* and reduced levels of p‐STAT3 in the liver tissue of rats. The increase in PGC‐1α suggests improved mitochondrial health and function. Concurrently, the decrease in p‐STAT‐3 levels may indicate a reduction in the inflammatory burden, as p‐STAT‐3 is involved in mediating cellular responses to liver damage and inflammation [53, 54]. These findings align with those of El‐Ghannam et al. [23], who also reported that the modulation of these pathways by Lina contributes to enhanced mitochondrial function and reduced inflammation in liver tissue.

These findings have important clinical implications. The ability of Lina to protect against Cis‐induced hepatotoxicity without compromising its anticancer effects (as evidenced by previous studies) suggests its potential as an adjuvant therapy in cancer treatment regimens involving Cis. By mitigating liver damage, Lina could potentially allow for more aggressive chemotherapy regimens or extended treatment durations, potentially improving cancer treatment outcomes. We acknowledge the absence of a Lina‐only control group in the present study. While our primary aim was to assess Lina’s protective effect against Cis‐induced injury, the inclusion of a group treated with Lina alone would have allowed us to definitively rule out any potential intrinsic hepatotoxicity or off‐target effects of Lina itself under our specific experimental conditions. Although Lina is generally considered safe and the hepatoprotective effect observed is robust when comparing the Cis and Cis + Lina groups, the lack of this control means we cannot empirically confirm the absence of such effects in this model.

We acknowledge published clinical reports linking Lina to potential drug‐induced liver injury (DILI). These include a monotherapy case with hepatocellular injury arising ∼4 weeks after initiation and improving upon withdrawal (Naranjo 6; RUCAM 7) [55], a biopsy‐supported cholestatic/cytolytic case with worsening on reexposure and resolution after discontinuation [56], and a report describing hepatitis after Lina with recurrence on sitagliptin, suggesting a possible DPP‐4 class effect [57]. Consistent with these isolated cases, LiverTox classifies Lina as a possible, rare cause of clinically apparent acute liver injury (likelihood score D), with typical latency of 1–4 weeks and a generally self‐limited course after drug cessation [58]. Accordingly, while our preclinical data support hepatoprotection in the Cis model, clinical translation should include careful hepatic monitoring and consideration of DILI risk. Although early observational signals and FDA Adverse Event Reporting System (FAERS) data raised concerns about a possible link between DPP‐4 inhibitors (including Lina) and increased pancreatic cancer risk, more robust evidence including a meta‐analysis of 165 randomized trials found no significant association (OR 0.86; 95% CI, 0.60–1.24) with pancreatic cancer or pancreatitis [59]. Our finding of hepatoprotection by Lina in a short‐term rat model of Cis‐induced toxicity cannot address long‐term oncogenic risks, which may stem from complex, tissue‐specific DPP‐4–mediated pathways and confounding in human diabetes populations; hence, the results must not be overgeneralized.

## 5. Conclusion

The present investigation supports that Lina shields the liver from Cis‐induced toxicity. Specifically, Lina at a dose of 10 mg/kg showed protective effects against Cis‐induced liver damage, which acts through numerous mechanisms by decreasing hepatic enzymes and lipid molecules, including the enhancement of antioxidative defenses, suppression of inflammatory cytokine production, and improvement of mitochondrial function. The protective effects were achieved through modulation of the STAT3/SIRT1/AMPK/PGC‐1α signaling pathway, as evidenced by the upregulation of SIRT1 and PGC‐1α, and the downregulation of phosphorylated STAT3. These beneficial effects, however, were not skewed toward specific outcomes. As soon as corrections were made, the enzyme levels decreased, and the hepatic architecture was relatively preserved, incorporating reduced expression of fibrotic markers such as TGF‐β and α‐SMA, as determined by SIRT1/PGC‐1α Western blot analysis. With its ability to mitigate Cis‐induced hepatotoxicity and potentiate Cis‐based cancer therapy, Lina appears to be a promising candidate for supportive treatment in cancer therapy. These results can be used to develop more effective management strategies for protecting patients from the effects of chemotherapy, household hepatotoxicity, and improving the treatment of oncological diseases. From a translational perspective, these findings open several avenues for future research: (1) long‐term studies in animal models should evaluate whether Lina’s hepatoprotective effects are sustained during repeated Cis cycles; (2) in vitro studies using hepatic cell lines and cancer cell cocultures are needed to confirm that Lina does not interfere with Cis’s anticancer efficacy; (3) pharmacokinetic studies should assess potential drug–drug interactions between Lina and Cis; and (4) clinical pilot studies in cancer patients receiving Cis‐based regimens could evaluate Lina as an adjuvant hepatoprotective agent, with careful monitoring of liver function and chemotherapy outcomes. Such studies would help translate our preclinical findings into clinical practice and potentially improve the therapeutic index of Cis‐based chemotherapy. However, it is necessary to conduct additional studies on patients to verify the aforementioned protective characteristics.

## 6. Study Limitation

This study has several limitations that should be considered. First, since the research was conducted using a rat model, the findings may not be directly applicable to patients until they are validated clinically.

Second, regarding study design, the trial examined only a single dose of Cis (8 mg/kg) and two doses of Lina (5 and 10 mg/kg). While this provides valuable preliminary insights, it limits the understanding of dose–response relationships for both Cis toxicity and Lina’s protective effects. Future studies incorporating broader dose ranges are needed to confirm and optimize the therapeutic effects.

Third, a Lina‐only control group was not included. Such a control is important for ruling out any potential hepatotoxicity or off‐target effects of Lina itself. The absence of this group means we cannot empirically rule out subtle, independent effects of Lina on the liver, although the protective effect observed between the Cis and Cis + Lina groups remains robust.

Fourth, the sample size of six rats per group (*n* = 6), while acceptable and standard for ethical preclinical studies to detect major effects, may limit the statistical power to detect more subtle changes in the measured parameters. Consequently, the findings should be interpreted within the context of this limitation, and further studies with larger cohorts are recommended to validate the results.

Fifth, the research did not investigate the influence of Lina on the effectiveness of Cis as an anticancer agent, nor did it include any in vitro tests to validate the mechanism. Additionally, the research did not investigate the impacts of delayed toxicities or long‐term hepatoprotective effects.

To enhance the therapeutic potential of Lina in reducing Cis‐induced hepatotoxicity, it is essential to conduct additional dose‐optimization, long‐term, and clinical trials.

**Nomenclature**
ADP: Adenosine diphosphate
ALT: Alanine aminotransferase
AMPK: AMP‐activated protein kinase
AST: Aspartate aminotransferase
ATP: Adenosine triphosphate
Cis: Cisplatin
DAB: 3,3′‐Diaminobenzidine tetrahydrochloride
DAPI: 4′,6‐Diamidino‐2‐phenylindole
DPP‐4: Dipeptidyl peptidase‐4
ELISA: Enzyme‐linked immunosorbent assay
ERK: Extracellular signal‐regulated kinase
FDA: Food and drug administration
GLP‐1: Glucagon‐like peptide‐1
GSH: Glutathione
H&E: Hematoxylin and eosin
HRP: Horseradish peroxidase
IL‐6: Interleukin‐6
IL‐10: Interleukin‐10
IP: Intraperitoneal
Lina: Linagliptin
MDA: Malondialdehyde
NADP: Nicotinamide adenine dinucleotide phosphate
NADPH: Nicotinamide adenine dinucleotide phosphate (reduced)
NF‐κB: Nuclear factor kappa B
NRC: National research centre
PBS: Phosphate‐buffered saline
PGC‐1α: Peroxisome proliferator‐activated receptor gamma coactivator 1‐alpha
ROS: Reactive oxygen species
SEM: Standard error of the mean
SIRT1: Silent mating‐type information regulation 2 homolog 1
SOD: Superoxide dismutase
STAT3: Signal transducer and activator of transcription 3
TAG: Triacylglycerol
TC: Total cholesterol
TGF‐β1: Transforming growth factor beta 1
TNF‐α: Tumor necrosis factor alpha
α‐SMA: Alpha‐smooth muscle actin

## Author Contributions

Marawan A. Elbaset, Bassim M. S. A. Mohamed, Passant E. Moustafa, Yosra Assem Hussien, and Hany M. Fayed: conceptualization, data curation, formal analysis, investigation, methodology, software, validation, visualization, writing–original draft, and writing–review and editing. Zeinab A. El‐Gendy: writing–original draft. Sherif M. Afifi: Visualization and writing–review and editing. Tuba Esatbeyoglu: methodology, review and editing, and funding acquisition. Alyaa Farouk Hessin: conceptualization, data curation, investigation, methodology, supervision, validation, visualization, writing–original draft, and writing–review and editing. Reda Korany M. S.: methodology, software, validation, visualization, writing–original draft, and writing–review and editing.

## Funding

The authors have nothing to report.

Open Access funding enabled and organized by Projekt DEAL.

## Conflicts of Interest

The authors declare no conflicts of interest.

## Supporting Information

Additional supporting information can be found online in the Supporting Information section.

## Supporting information


**Supporting Information 1** Supporting Figure 1: The western blot assay of PGC1 alpha protein expression (original blots).

**Supporting Information 2** Supporting Figure 2: The western blot assay of SIRT1 protein expression (original blots).

**Supporting Information 3** Supporting Figure 3: The western blot assay of β‐actin protein expression (original blots).

## Data Availability

The data that support the findings of this study are available upon reasonable request.
