# Supplementary material for: Protective Role of Linagliptin in Cisplatin‐Mediated Liver Injury: Involvement of STAT3 and AMPK/SIRT1/PGC‐1alpha Mitochondrial Energy Sensing Networks
Source: Adv Pharmacol Pharm Sci. 2026 Jul 7;2026:6457551. doi: 10.1155/adpp/6457551 (PMC13342701; doi:10.1155/adpp/6457551)
Supplement: Supplementary file 1 — Supporting Information 1 Supporting Figure 1: The western blot assay of PGC1 alpha protein expression (original blots). [file ADPP-2026-6457551-s001.docx]

**Western blot analysis of protein expression (original blots)**

**
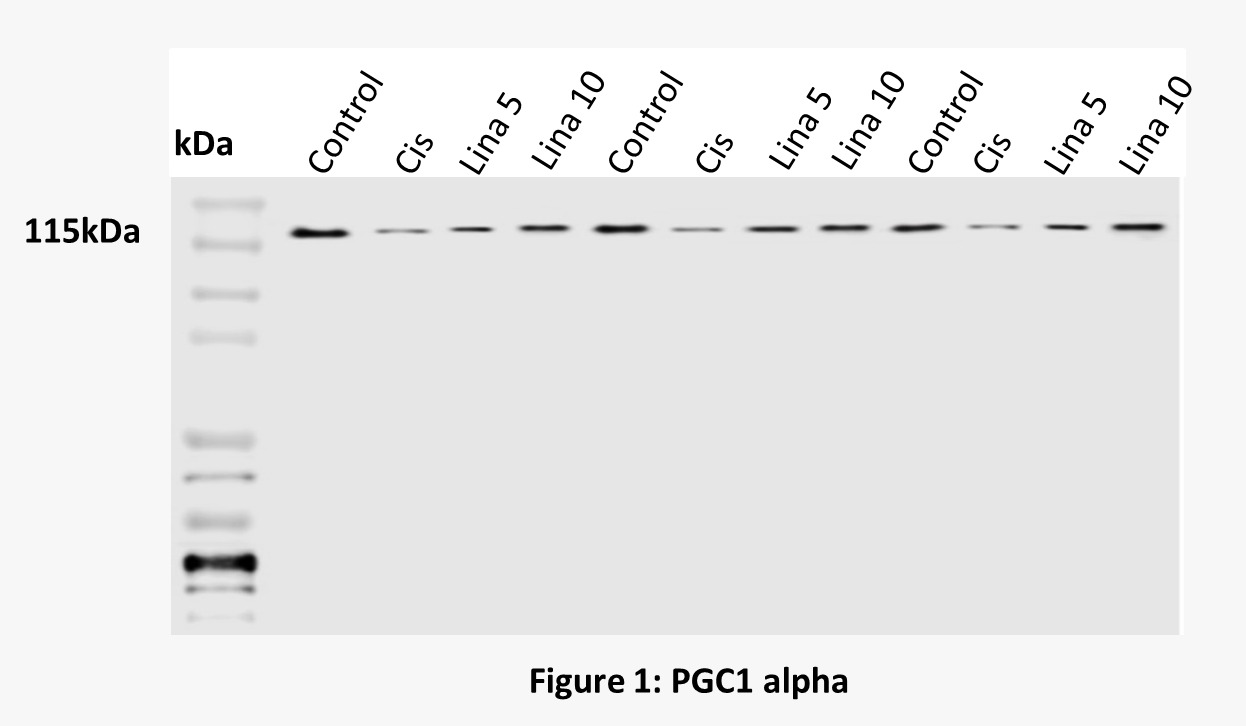
**

**Figure 1:** The western blot assay of ‎ PGC1 alpha protein expression (original blots).
