# Supplementary material for: Protective Role of Linagliptin in Cisplatin‐Mediated Liver Injury: Involvement of STAT3 and AMPK/SIRT1/PGC‐1alpha Mitochondrial Energy Sensing Networks
Source: Adv Pharmacol Pharm Sci. 2026 Jul 7;2026:6457551. doi: 10.1155/adpp/6457551 (PMC13342701; doi:10.1155/adpp/6457551)
Supplement: Supplementary file 2 — Supporting Information 2 Supporting Figure 2: The western blot assay of SIRT1 protein expression (original blots). [file ADPP-2026-6457551-s003.docx]

**Figure 2:‎ Sirt1**


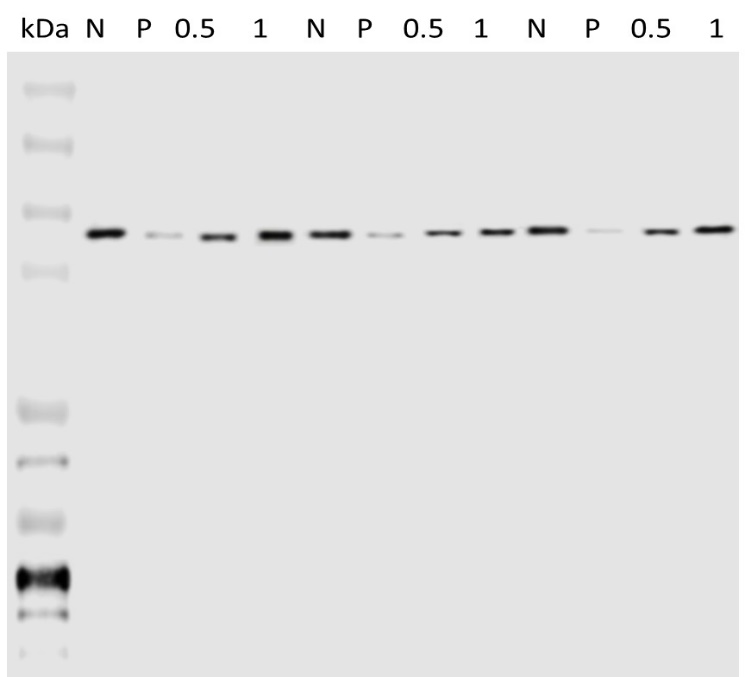


**kDa**

Control

Cis

Lina 5

Lina 10

Control

Cis

Lina 5

Lina 10

Lina 10

Cis

Lina 5

Control

**81 kDa**

**Figure 2:** The western blot assay of ‎ Sirt1 protein expression (original blots).
