# Supplementary figures and images for: Protective Role of Linagliptin in Cisplatin‐Mediated Liver Injury: Involvement of STAT3 and AMPK/SIRT1/PGC‐1alpha Mitochondrial Energy Sensing Networks
Source: Adv Pharmacol Pharm Sci. 2026 Jul 7;2026:6457551. doi: 10.1155/adpp/6457551 (PMC13342701; doi:10.1155/adpp/6457551)

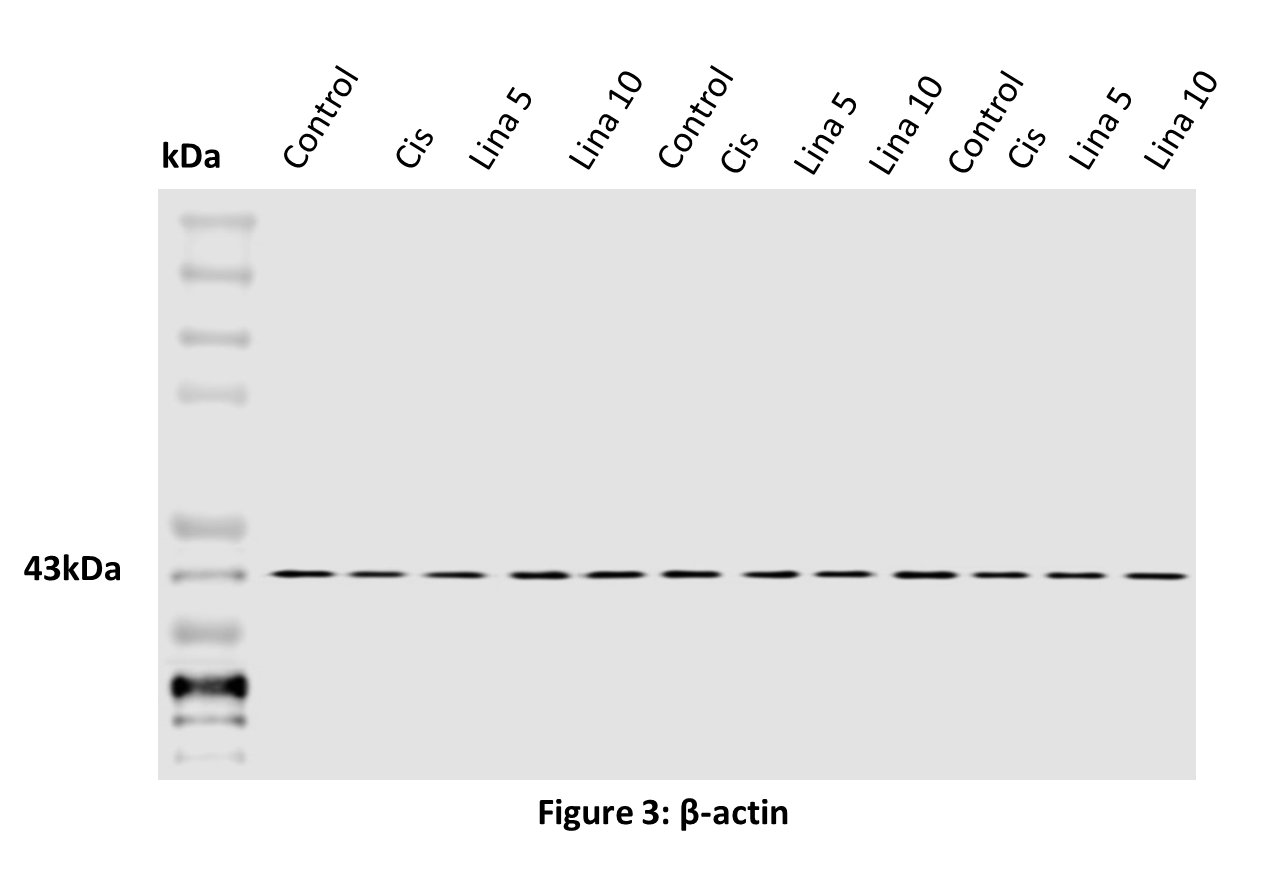

**Figure 3:** The western blot assay of ‎ β-actin protein expression (original blots).

Supplement: Supplementary file 3 — Supporting Information 3 Supporting Figure 3: The western blot assay of β‐actin protein expression (original blots). [file ADPP-2026-6457551-s002.docx]
